# PPP2R2C confers radioresistance in nasopharyngeal carcinoma by suppressing ferroptosis via RPS27L stabilization

**DOI:** 10.1038/s41419-026-08732-y

**Published:** 2026-05-11

**Authors:** Jianbo Fang, Qi Yang, Luxi Yang, Huiying Liu, Shulu Hu, Weitao Shen, Yueming Zhang, Jie Shen, Ting Wei, Qiong Lyu, Peng Luo, Xiaowen Wu, Jian Zhang, Hui Meng

**Affiliations:** 1 Department of Oncology, Zhujiang Hospital, Southern Medical University, Guangzhou, Guangdong China; 2 Department of Radiation Oncology, The Affiliated Cancer Hospital of Xiangya School of Medicine, Central South University/Hunan Cancer Hospital, Changsha, Hunan China; 3 Department of Oncology, Da Feng hospital, Shantou, Guangdong China

**Keywords:** Radiotherapy, Oncogenes

## Abstract

Radiotherapy resistance remains a major obstacle in nasopharyngeal carcinoma (NPC). This study delineates the role of protein phosphatase 2 regulatory subunit B gamma (PPP2R2C) in NPC radioresistance and its underlying mechanism to identify therapeutic targets. Through integrated bioinformatic analysis, PPP2R2C was identified as a candidate radioresistance driver. In vitro and in vivo functional assays demonstrated that PPP2R2C depletion significantly impaired NPC cell proliferation, migration, and radioresistance, while its overexpression enhanced these phenotypes. Mechanistic investigations revealed PPP2R2C inhibits radiation-induced ferroptosis, evidenced by transmission electron microscopy (TEM), lipid reactive oxygen species (ROS) quantification, malondialdehyde (MDA) assays, and immunoblotting of glutathione peroxidase 4 (GPX4) and solute carrier family 7 member 11 (SLC7A11). Crucially, immunoprecipitation-mass spectrometry (IP-MS), co-immunoprecipitation (Co-IP), and immunofluorescence (IF) confirmed PPP2R2C physically interacts with RPS27L. Further analysis via qPCR, Western blotting, cycloheximide chase, and proteasome inhibition showed PPP2R2C stabilizes RPS27L protein by blocking proteasomal degradation. RPS27L knockdown reversed PPP2R2C-mediated radioresistance and ferroptosis suppression. Clinically, high PPP2R2C expression correlated with poor patient survival. These findings establish that PPP2R2C promotes NPC radioresistance by stabilizing RPS27L to inhibit ferroptosis, positioning the PPP2R2C–RPS27L axis as a novel prognostic biomarker and therapeutic target for overcoming radioresistance.

## Introduction

Prevalent in southern China, Southeast Asia, and North Africa, nasopharyngeal carcinoma (NPC) exhibits relatively high radiosensitivity among solid tumors, establishing radiotherapy as one of its main treatment modalities [1–3]. Intensity-modulated radiotherapy has improved the 5-year overall survival rate for NPC patients to more than 80%. Nevertheless, 10–20% of patients still develop local recurrence or distant metastasis following definitive treatment [4]. Accumulating evidence indicates that most recurrences are in-field and arise within the high-dose radiation field [5], suggesting that conventional radiotherapy doses are insufficient to eradicate all tumor cells in the primary lesion. The repopulation of residual tumor cells subsequently leads to the formation of recurrent foci, underscoring radioresistance as a major obstacle to successful NPC radiotherapy [6–8]. Consequently, elucidating the novel molecular mechanisms underlying radioresistance and exploring effective strategies to enhance the therapeutic response represent critical research priorities for improving the prognosis of NPC patients [9].

Radioresistance in NPC is driven by a multitude of mechanisms, including EBV involvement, cancer stem cell properties, enhanced reactive oxygen species (ROS) scavenging, augmented DNA damage repair capacity, dysregulated programmed cell death, and remodeling of the tumor microenvironment [10–12]. In particular, ferroptosis, an iron-dependent form of regulated cell death triggered by excessive lipid peroxidation [13], has attracted considerable interest due to its dual function in modulating intrinsic radioresistance and serving as a tractable target for radiosensitization [14]. Notably, NPC cells exploit multiple strategies to evade radiotherapy-induced ferroptosis, thereby acquiring resistance [15–18]. For instance, these cells upregulate key ferroptosis defense proteins such as glutathione peroxidase 4 (GPX4) and solute carrier family 7 member 11 (SLC7A11), which reduce intracellular lipid peroxidation and promote cell survival following irradiation [15, 16, 19]. Zhou et al. reported that the histone deacetylase 2 (HDAC2)/sirtuin 3 (SIRT3) axis mediates ACSL4 acetylation and overexpression in NPC; importantly, ACSL4 silencing suppressed ferroptosis and thus conferred radioresistance [20]. Conversely, boosting ferroptosis represents a promising strategy to overcome radioresistance. Glutathione S-transferase mu 3 (GSTM3) promotes ferroptosis and radiosensitivity by stabilizing the ubiquitin-specific protease 14 (USP14)–fatty acid synthase (FASN) axis (thereby inhibiting FASN ubiquitination and degradation) while concurrently interacting with and suppressing GPX4 expression [16]. Furthermore, synthetic cell membrane-coated semiconductor polymeric nanoparticles co-delivering iron ions and circADARB1-targeting small interfering RNAs (siRNAs) synergistically trigger ferroptosis and effectively improve NPC radiosensitivity [21]. Collectively, these findings underscore the pivotal role of ferroptosis in NPC radiotherapy and its substantial translational potential.

Protein phosphatase 2 regulatory subunit B gamma (PPP2R2C) encodes the B55γ protein, a regulatory B subunit of protein phosphatase 2A (PP2A) [22]. This subunit exists within cells both in a free form and as part of the PP2A holoenzyme [23], and is critically involved in governing the subcellular localization and substrate recognition of PP2A [24]. Previous studies have identified PPP2R2C as a potential tumor suppressor in prostate cancer and ovarian cancer [25, 26]. In glioma, PPP2R2C exhibits dual functions: it suppresses tumor cell growth by inhibiting the mTOR pathway [27], yet conversely promotes glioma cell survival under glucose-deprived conditions by upregulating salt-inducible kinase 2 (SIK2) [28]. Notably, recent research has linked PPP2R2C to neural cell senescence, as PPP2R2C knockout significantly elevated DNA damage levels in both zebrafish and mouse models [29]. However, the specific role of PPP2R2C in NPC remains unexplored, and its potential relationship with radioresistance has not been investigated.

Ribosomal protein S27L (RPS27L) encodes an evolutionarily conserved 84-amino-acid ribosomal protein belonging to the 40S small subunit. As a bona fide target of the p53–mouse double minute 2 homolog (MDM2) axis, RPS27L acts as an oncogenic driver that promotes radioresistance [30]. Studies show that RPS27L interacts with Fanconi anemia (FA) proteins, and silencing RPS27L significantly reduces intracellular FA protein levels and the activity of the DNA crosslink repair pathway [31]. RPS27L knockout also enhances mouse radiosensitivity to ionizing radiation via the MDM2–p53 and MDM2–MRN–ATM axes [32]. Additionally, in breast cancer and non-small cell lung cancer cells, the oncogene MDM2 stabilizes RPS27L protein through increased neddylation modification, thereby promoting cancer cell survival [33]. Currently, the role of RPS27L in NPC remains uncharacterized. Moreover, the mechanistic link between PPP2R2C and RPS27L in radioresistance remains unclear.

In this study, we identified PPP2R2C as a candidate radioresistance-associated gene through integrated bioinformatic analysis. This finding was further validated by a series of in vitro and in vivo experiments. Further investigation revealed that PPP2R2C promotes radioresistance in NPC cells by inhibiting ferroptosis. Mechanistically, PPP2R2C interacts with and stabilizes RPS27L by preventing its proteasomal degradation. Collectively, our work characterizes the pro-radioresistance function of PPP2R2C in NPC, identifies RPS27L as a critical downstream interactor, and delineates the essential role of the PPP2R2C–RPS27L axis in regulating ferroptosis, thus providing novel potential targets and therapeutic strategies for overcoming radioresistance in NPC.

## Results

### PPP2R2C is upregulated in NPC and correlated with poor prognosis

**Fig. 1:**
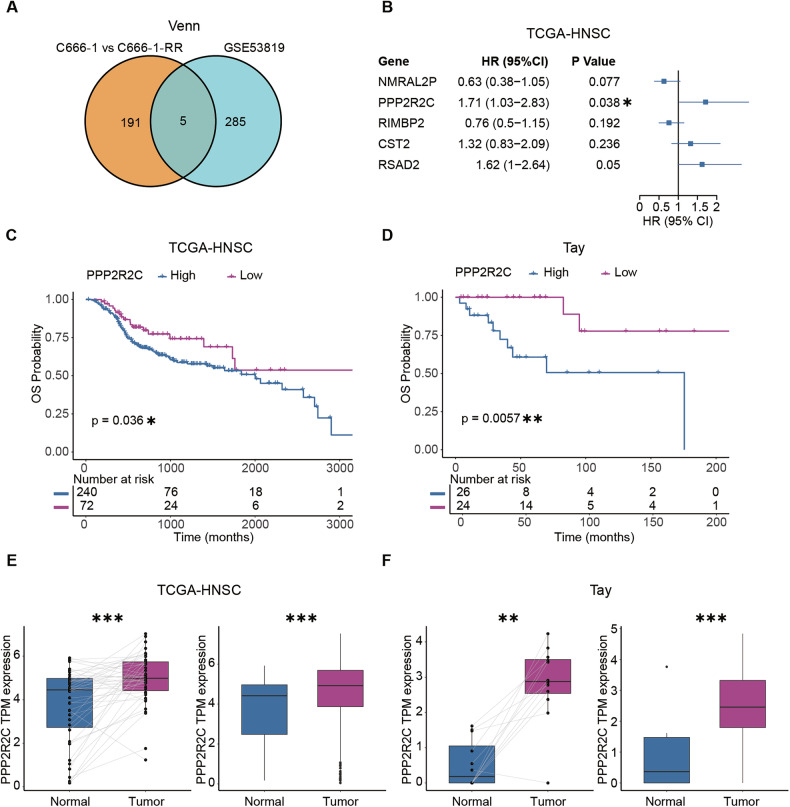
PPP2R2C upregulation correlates with poor NPC prognosis. **A** Venn diagram showing the co-expressed DEGs between cell lines and patient tissues. **B** Univariate Cox regression identifies PPP2R2C as a significant risk factor in TCGA-HNSC cohort. **C**, **D** KM survival curves demonstrate significantly worse OS with high PPP2R2C expression in TCGA-HNSC and Tay cohort. **E**, **F** Elevated PPP2R2C mRNA expression in NPC tumor tissues versus normal tissues. TCGA-HNSC, The Cancer Genome Atlas Head and Neck Squamous Cell Carcinoma. KM, Kaplan–Meier. OS, Overall survival. DEGs, differentially expressed genes. ^*^P < 0.05, ^**^P < 0.01, ^***^P < 0.001.

In our previous study, we successfully established two radioresistant NPC cell lines, C666-1-RR and HONE1-RR, derived from parental C666-1 and HONE1 cell lines using fractionated irradiation (IR) [34]. We performed high-throughput RNA sequencing on C666-1 and C666-1-RR cells. Differential gene expression analysis against both cell dataset and public dataset (GSE53819) identified five shared differentially expressed genes (DEGs): NMRAL2P, PPP2R2C, RIMBP2, CST2, and RSAD2 (Fig. 1A). Univariate Cox regression analysis of these five DEGs within the TCGA-HNSC cohort revealed that only PPP2R2C expression served as a significant poor prognostic factor (Fig. 1B). Kaplan–Meier (KM) survival analysis performed on both the TCGA-HNSC and Tay cohorts [35] consistently demonstrated that high PPP2R2C expression was significantly associated with poor overall survival (OS) (Fig. 1C, D). Additionally, expression analysis across both cohorts further revealed significantly higher PPP2R2C levels in tumor tissues compared to normal tissues, irrespective of paired or unpaired analytical methods (Fig. 1E, F). Collectively, these findings implicate PPP2R2C in NPC malignant progression and radioresistance, highlighting its potential as a prognostic biomarker for radiotherapy resistance.

### PPP2R2C promotes NPC cells proliferation, migration and radioresistance

**Fig. 2:**
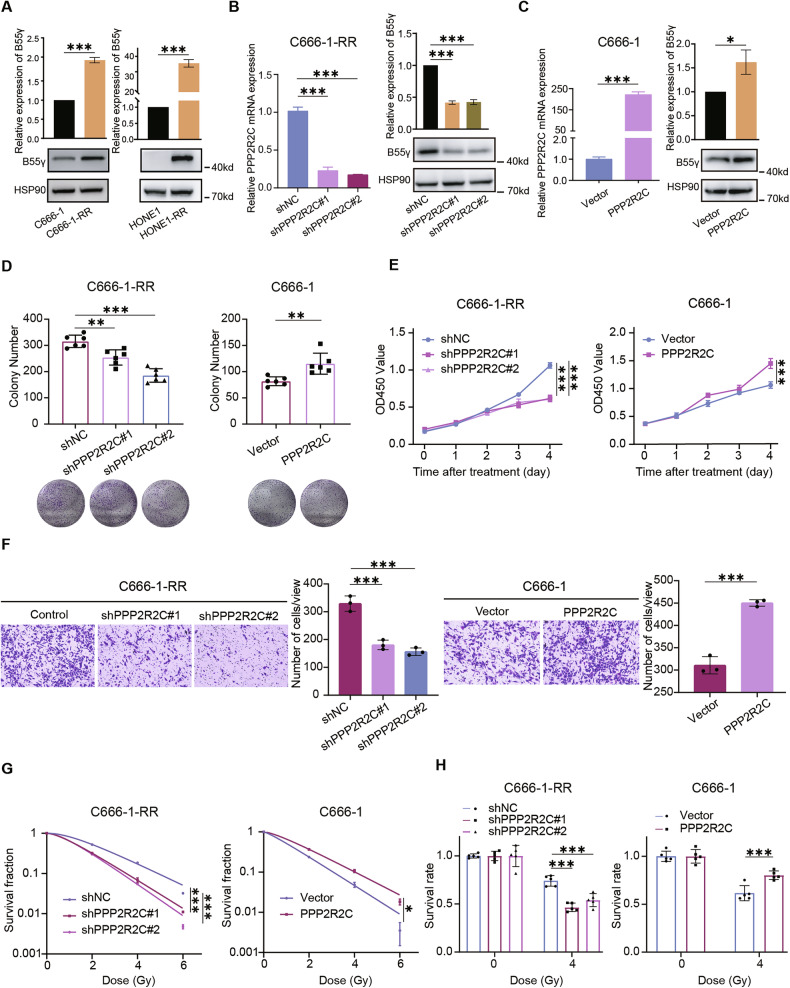
PPP2R2C drives malignant phenotypes and radioresistance. **A** Upregulated B55γ (the protein encoded by PPP2R2C) in C666-1-RR and HONE1-RR cell lines. *n* = 3 per group. Data are shown as mean ± SD. **B** Reduced PPP2R2C mRNA/protein post-knockdown in C666-1-RR cells. *n* = 3 per group. Data are shown as mean ± SD. **C** Increased PPP2R2C mRNA/protein after overexpression in C666-1 cells. *n* = 3 per group. Data are shown as mean ± SD. **D** Reduced colony formation with PPP2R2C knockdown in C666-1-RR cells; increased colony formation with overexpression in C666-1 cells. *n* = 6 per group. Data are shown as mean ± SD. **E** Impaired proliferation with PPP2R2C knockdown in C666-1-RR cells; enhanced proliferation with overexpression in C666-1 cells. *n* = 3 per group. Data are shown as mean ± SD. **F** Decreased migration with PPP2R2C knockdown in C666-1-RR cells; increased migration with overexpression in C666-1 cells. *n* = 3 per group. Data are shown as mean ± SD. **G** Reduced radioresistance with PPP2R2C knockdown in C666-1-RR cells; increased radioresistance with overexpression in C666-1 cells. *n* = 3 per group. Data are shown as mean ± SD. **H** Decreased post-4 Gy IR viability with PPP2R2C knockdown in C666-1-RR cells; increased viability with overexpression in C666-1 cells. *n* = 3 per group. Data are shown as mean ± SD. IR, irradiation. Statistical significance was evaluated using two-tailed independent Student’s *t*-test or one-way ANOVA with Dunnett’s multiple comparisons test. ^*^P < 0.05, ^**^P < 0.01, ^***^P < 0.001.

Next, we conducted further experimental investigations. Western blotting analysis revealed significantly higher expression levels of B55γ (encoded by PPP2R2C) in radioresistant cell lines compared to their parental counterparts (Fig. 2A). To functionally characterize the role of PPP2R2C in radioresistance, we generated NPC cells with stable knockdown of PPP2R2C (shPPP2R2C) or PPP2R2C overexpression. Modulation efficiency was confirmed by RT-qPCR and Western blotting (Figs. 2B, C and S1A, B). Functional assessment revealed that PPP2R2C knockdown suppressed malignant phenotypes, including clonogenic capacity (Figs. 2D and S1C), proliferation (Figs. 2E and S1D), and migration (Figs. 2F and S1E), whereas overexpression enhanced these behaviors. Notably, PPP2R2C depletion radiosensitized NPC cells, as evidenced by reduced clonogenic survival after IR (Figs. 2G and S1F). Consistent with this, CCK-8 assays demonstrated decreased viability in irradiated shPPP2R2C cells but increased viability in PPP2R2C-overexpressing cells (Figs. 2H and S1G). These data collectively establish PPP2R2C as a promoter of tumor aggressiveness and radioresistance in NPC.

### PPP2R2C promotes radioresistance via suppressing ferroptosis in NPC cells

**Fig. 3:**
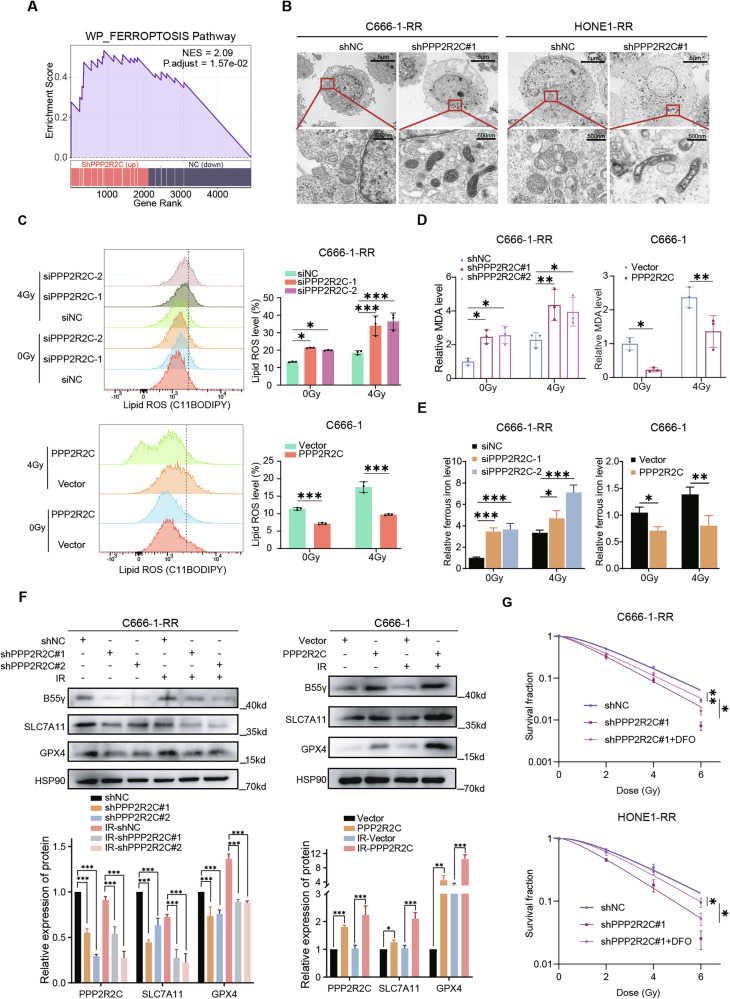
PPP2R2C inhibits ferroptosis to promote radioresistance. **A** Pathway enrichment analyses following PPP2R2C knockdown in C666-1-RR cells. **B** TEM showing mitochondrial alterations induced by PPP2R2C knockdown in C666-1-RR and HONE1-RR cells. **C** Lipid ROS, **D** MDA and **E** intracellular ferrous iron measurements in PPP2R2C-knockdown C666-1-RR cells and PPP2R2C-overexpressing C666-1 cells. *n* = 3 per group. Data are shown as mean ± SD. **F** Changes in protein expression of SLC7A11 and GPX4 in PPP2R2C-knockdown C666-1-RR cells and PPP2R2C-overexpressing C666-1 cells with or without IR. *n* = 3 per group. Data are shown as mean ± SD. **G** DFO rescued PPP2R2C knockdown-induced radiosensitivity in C666-1-RR and HONE1-RR cells. *n* = 3 per group. Data are shown as mean ± SD. IR, irradiation. TEM, Transmission electron microscopy. ROS, reactive oxygen species. MDA, malondialdehyde. DFO, deferoxamine mesylate. Scale bars: 5 μm, 500 nm. Statistical significance was evaluated using two-tailed independent Student’s *t*-test or one-way ANOVA with Dunnett’s multiple comparisons test. ^*^P < 0.05, ^**^P < 0.01, ^***^P < 0.001.

To elucidate the mechanism by which PPP2R2C promotes radioresistance in NPC, we performed high-throughput RNA sequencing on C666-1-RR cells following PPP2R2C knockdown. Enrichment analysis revealed significant upregulation of the WP_FERROPTOSIS_Pathway in PPP2R2C knockdown cells (Fig. 3A). Given the established role of ferroptosis in modulating radiotherapy response, we hypothesized that PPP2R2C confers radioresistance by suppressing ferroptosis. Transmission electron microscopy (TEM) demonstrated that PPP2R2C knockdown induced characteristic ferroptotic mitochondrial alterations, including increased mitochondrial membrane density, reduced mitochondrial volume, disappearance of cristae, and outer membrane rupture (Fig. 3B). As lipid peroxidation is a defining feature of ferroptosis, we quantified lipid reactive oxygen species (ROS) by flow cytometry (Figs. 3C and S2A) and malondialdehyde (MDA) levels—a key lipid peroxidation byproduct—using the thiobarbituric acid (TBA) assay (Figs. 3D and S2B). PPP2R2C knockdown in radioresistant lines significantly increased both lipid ROS and MDA levels, irrespective of IR status. Conversely, PPP2R2C overexpression in radiosensitive lines suppressed lipid peroxidation. Given that ferrous iron (Fe²⁺) accumulation is an initial and essential step in ferroptosis, we quantified intracellular Fe²⁺ levels using a ferrous iron assay kit (Figs. 3E and S2C). PPP2R2C knockdown significantly increased Fe²⁺ levels in radioresistant cells, irrespective of IR status, while PPP2R2C overexpression had the opposite effect in radiosensitive cells. Western blotting analysis further confirmed consistent regulation of key ferroptosis defense proteins: SLC7A11 and GPX4 expression decreased post-PPP2R2C knockdown but increased upon overexpression, independent of IR exposure (Figs. 3F and S2D). Critically, the compromised radioresistance phenotype observed in PPP2R2C-knockdown cells after IR was rescued by the ferroptosis inhibitors deferoxamine mesylate (DFO) and ferrostatin-1 (Fer-1), as evidenced by restored clonogenic survival (Figs. 3G and S2E). Collectively, these results demonstrate that PPP2R2C promotes radioresistance in NPC cells by inhibiting ferroptosis.

### RPS27L interacts with PPP2R2C to collectively mediate radioresistance in NPC cells

**Fig. 4:**
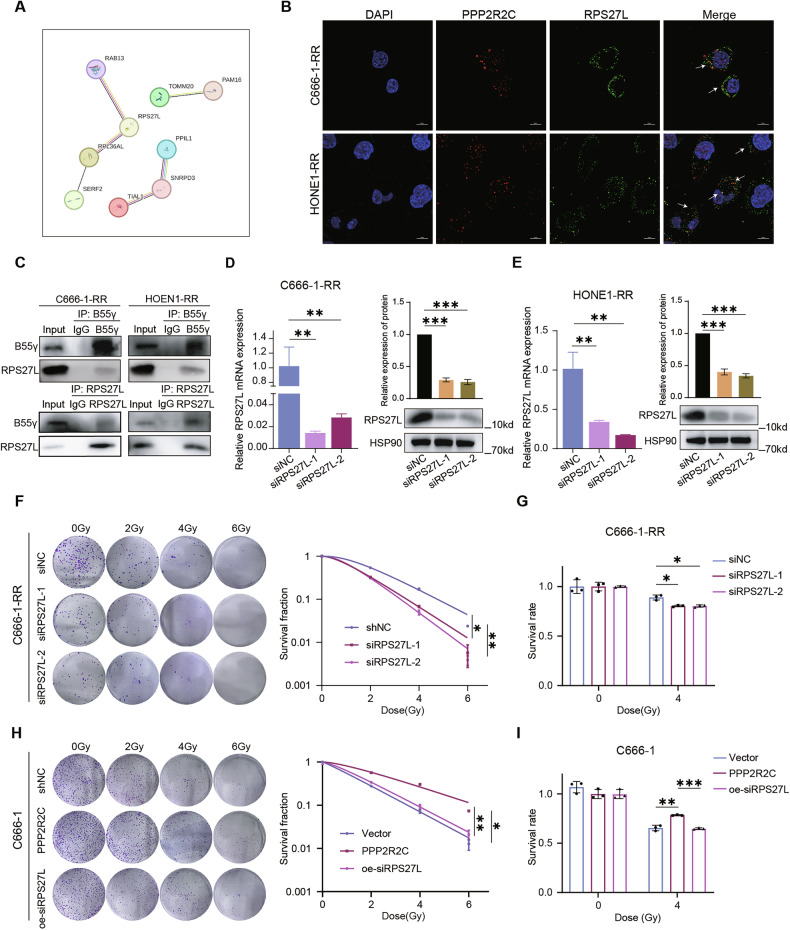
PPP2R2C–RPS27L interaction mediates radioresistance. **A** RPS27L was identified as the top interactor of B55γ in PPP2R2C-overexpressing HONE1 cells. **B** IF assays and **C** Co-IP assays demonstrated the interaction between RPS27L and B55γ. Efficient RPS27L knockdown at mRNA/protein levels in **D** C666-1-RR cells and **E** HONE1-RR cells. *n* = 3 per group. Data are shown as mean ± SD. RPS27L knockdown impaired **F** clonogenic survival and **G** viability following 4 Gy IR in C666-1-RR cells. *n* = 3 per group. Data are shown as mean ± SD. **H**, **I** RPS27L knockdown reversed PPP2R2C-overexpression-induced radioresistance in C666-1 cells. *n* = 3 per group. Data are shown as mean ± SD. IR, irradiation. Co-IP, Co-immunoprecipitation. IF, immunofluorescence. Scale bars: 10 μm. Statistical significance was evaluated using two-tailed independent Student’s *t*-test or one-way ANOVA with Dunnett’s multiple comparisons test. ^*^P < 0.05, ^**^P < 0.01, ^***^P < 0.001.

Given the substrate recognition specificity of B55γ, we performed immunoprecipitation-mass spectrometry (IP-MS) in PPP2R2C-overexpressing HONE1 cells to delineate its radioresistance mechanism. The top 15 candidate proteins were analyzed using the publicly available STRING database (version 12.0; https://string-db.org/), which identified RPS27L as the central hub protein (Fig. 4A). Immunofluorescence (IF) demonstrated subcellular colocalization of PPP2R2C and RPS27L (Fig. 4B), while reciprocal co-immunoprecipitation (Co-IP) experiments confirmed their specific physical interaction in both radioresistant lines (Fig. 4C). For the forward pull-down assay, anti-PPP2R2C antibody was used for immunoprecipitation (IP), followed by Western blotting with anti-RPS27L antibody to detect co-precipitated RPS27L. For the reverse pull-down assay, anti-RPS27L antibody was employed for IP, and Western blotting with anti-PPP2R2C antibody identified co-precipitated PPP2R2C, excluding non-specific binding artifacts. Subsequent transient RPS27L knockdown in HONE1-RR and C666-1-RR cells (validated by RT-qPCR and Western blotting in Fig. 4D, E), significantly impaired post-IR clonogenic survival and cell viability (Figs. 4F, G and S3C, D). To further confirm the oncogenic role of RPS27L in radioresistance, we transiently overexpressed RPS27L in radiosensitive parental C666-1 and HONE1 cells (Fig. S3A, B). RPS27L overexpression alone was sufficient to enhance clonogenic survival and cell viability following IR exposure (Fig. S3E–H). Crucially, RPS27L depletion reversed the radioresistance conferred by PPP2R2C overexpression in parental NPC cells (Figs. 4H, I and S3I, J). These results establish that RPS27L physically and functionally cooperates with PPP2R2C to promote radioresistance in NPC.

### RPS27L is stabilized by PPP2R2C and contributes to the inhibition of cellular ferroptosis

**Fig. 5:**
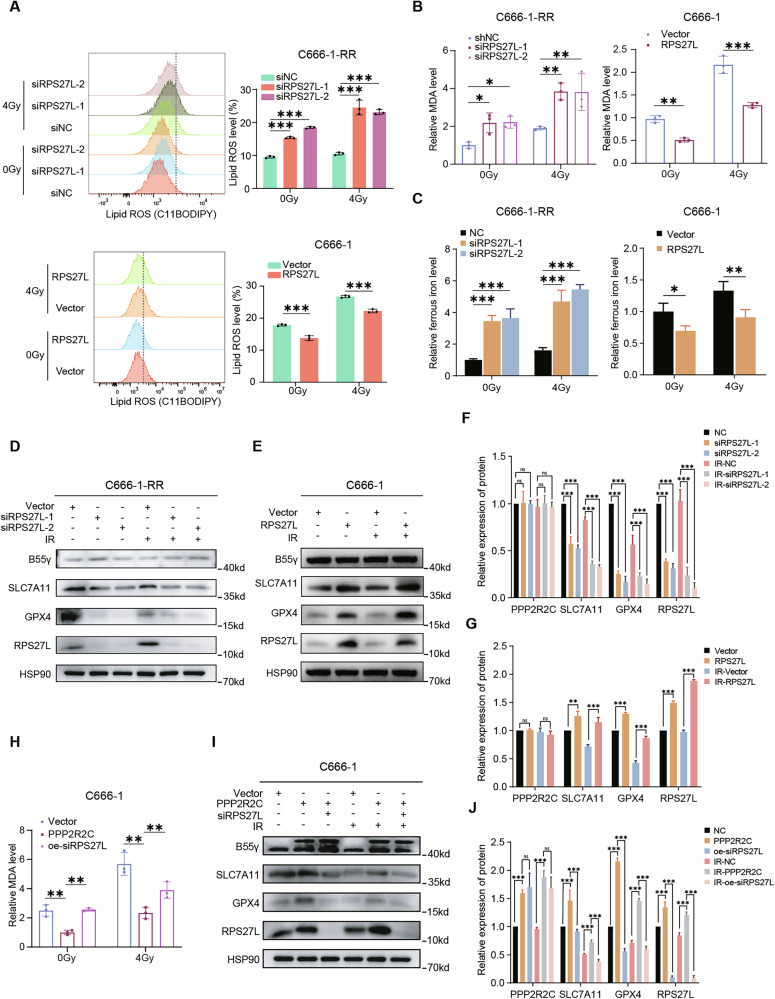
PPP2R2C inhibits ferroptosis via RPS27L. **A** Increased lipid ROS levels in RPS27L-knockdown C666-1-RR cells ± 4 Gy IR and decreased lipid ROS levels in RPS27L-overexpressing C666-1 cells ± 4 Gy IR. *n* = 3 per group. Data are shown as mean ± SD. **B** Increased MDA levels in RPS27L-knockdown C666-1-RR cells ±4 Gy IR and decreased MDA levels in RPS27L-overexpressing C666-1 cells ±4 Gy IR. *n* = 3 per group. Data are shown as mean ± SD. **C** Increased ferrous iron levels in RPS27L-knockdown C666-1-RR cells ±4 Gy IR and decreased ferrous iron levels in RPS27L-overexpressing C666-1 cells ±4 Gy IR. *n* = 3 per group. Data are shown as mean ± SD. **D** Western blot of SLC7A11/GPX4 protein expression after RPS27L knockdown ± IR in C666-1-RR cells. **E** Western blot of SLC7A11/GPX4 protein expression after RPS27L overexpression ± IR in C666-1 cells. **F** Statistical analyses of SLC7A11/GPX4 protein expression after RPS27L knockdown ± IR in C666-1-RR cells. *n* = 3 per group. Data are shown as mean ± SD. **G** Statistical analyses of SLC7A11/GPX4 protein expression after RPS27L overexpression ± IR in C666-1 cells. *n* = 3 per group. Data are shown as mean ± SD. RPS27L knockdown reverses PPP2R2C-overexpression-induced suppression of **H** MDA accumulation and **I**, **J** SLC7A11/GPX4 expression in C666-1 cells. *n* = 3 per group. Data are shown as mean ± SD. Statistical significance was evaluated using two-tailed independent Student’s *t*-test or one-way ANOVA with Dunnett’s multiple comparisons test. ^*^P < 0.05, ^**^P < 0.01, ^***^P < 0.001, ns: not significant.

Given RPS27L is a target of the p53–MDM2 axis (both regulate ferroptosis), we investigated whether RPS27L participates in PPP2R2C-mediated ferroptosis inhibition. Flow cytometry and MDA assays showed RPS27L knockdown increased lipid ROS and MDA in C666-1-RR/HONE1-RR cells regardless of IR, while RPS27L overexpression reduced their levels in parental cells (Figs. 5A, B and S4A, B). Consistent with the key role of ferrous iron Fe²⁺ in initiating ferroptosis, we quantified intracellular Fe²⁺ levels in C666-1-RR/HONE1-RR cells. RPS27L knockdown significantly increased Fe²⁺ accumulation, regardless of IR treatment. Conversely, transient overexpression of RPS27L in radiosensitive parental C666-1 and HONE1 cells significantly decreased Fe²⁺ levels (Figs. 5C and S4C). Ferroptosis suppressors SLC7A11 and GPX4 were downregulated upon RPS27L loss (Figs. 5D, F and S4D, F) and upregulated following RPS27L overexpression (Figs. 5E, G and S4E, G). Collectively, these data indicate that RPS27L inhibits ferroptosis in NPC cells. Rescue experiments (MDA assay and Western blotting) showed RPS27L knockdown reversed PPP2R2C-overexpression-enhanced ferroptosis resistance (Figs. 5H–J and S4H–J).

**Fig. 6:**
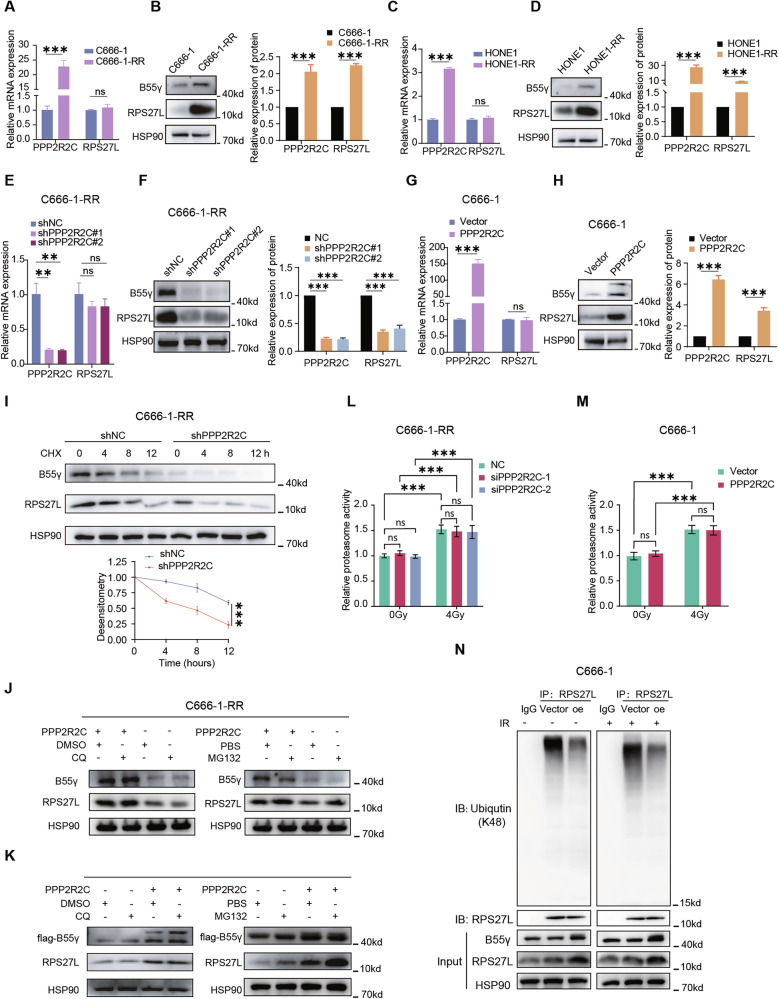
PPP2R2C stabilizes RPS27L by reducing its proteasomal degradation. **A**–**D** RPS27L mRNA/protein levels in parental and radioresistant paired cell lines (HONE1 vs. HONE1-RR; C666-1 vs. C666-1-RR). *n* = 3 per group. Data are shown as mean ± SD. RPS27L mRNA/protein levels following **E**, **F** PPP2R2C-knockdown in C666-1-RR cells and **G**, **H** PPP2R2C-overexpression in C666-1 cells. *n* = 3 per group. Data are shown as mean ± SD. **I** Cycloheximide (CHX) chase assay showed that PPP2R2C stabilized RPS27L protein in C666-1-RR cells. *n* = 3 per group. Data are shown as mean ± SD. Effects of chloroquine (CQ) and MG132 on RPS27L stability in **J** PPP2R2C-knockdown C666-1-RR cells and **K** PPP2R2C-overexpression C666-1 cells. *n* = 3 per group. Data are shown as mean ± SD. Changes in proteasomal activity in **L** C666-1-RR cells upon PPP2R2C knockdown and **M** C666-1 cells upon PPP2R2C overexpression. *n* = 3 per group. Data are shown as mean ± SD. **N** Ubiquitination (K48) assay of RPS27L performed in PPP2R2C-overexpressing C666-1 cells. CHX, cycloheximide. CQ, chloroquine. IR, irradiation. oe, PPP2R2C overexpression. Statistical significance was evaluated using two-tailed independent Student’s *t*-test or one-way ANOVA with Dunnett’s multiple comparisons test. ^*^P < 0.05, ^**^P < 0.01, ^***^P < 0.001, ns: not significant.

To further characterize the relationship between RPS27L and PPP2R2C, we measured their expression in parental and radioresistant NPC cell lines. Results showed that both transcriptional and protein levels of PPP2R2C, along with the protein level of RPS27L, were significantly higher in radioresistant strains compared to parental counterparts. Notably, RPS27L transcriptional levels exhibited no significant difference between the paired cell lines (Fig. 6A–D). We then assessed RPS27L expression following PPP2R2C knockdown or overexpression, finding no impact on RPS27L transcription but consistent changes in protein levels (Figs. 6E–H and S5A–D). Together, these data suggest that PPP2R2C regulates RPS27L protein levels via post-translational interaction. To validate this, cycloheximide (CHX) chase assay was conducted and showed PPP2R2C knockdown accelerated RPS27L degradation (Figs. 6I and S5E), confirming PPP2R2C stabilizes RPS27L protein.

To identify the specific degradation pathway of RPS27L, we performed inhibitor treatment experiments in both PPP2R2C-knockdown and overexpressing cells. In PPP2R2C-knockdown cells, MG132 (proteasome inhibitor) effectively restored RPS27L protein levels, whereas chloroquine (CQ, lysosome-autophagy inhibitor) had no obvious effect (Figs. 6J and S5F, K, L). Consistent results were obtained in PPP2R2C-overexpressing cells: MG132 treatment further increased RPS27L levels, while CQ remained ineffective (Figs. 6K and S5G, M, N). These data collectively demonstrate that RPS27L is degraded via the proteasomal pathway rather than the lysosome-autophagy pathway, and PPP2R2C modulates RPS27L stability by targeting this pathway.

We next measured proteasome activity using a fluorescence-based assay. As expected, IR treatment significantly increased proteasome activity in both parental and radioresistant NPC cells. However, neither PPP2R2C knockdown nor overexpression altered proteasome activity, regardless of IR exposure (Figs. 6L, M and S5H, I). This indicates that PPP2R2C does not modulate global proteasome function, but specifically regulates RPS27L degradation.

To dissect the molecular basis of PPP2R2C-mediated RPS27L stabilization, we examined K48-linked ubiquitination of RPS27L (the canonical signal for proteasomal degradation) via Co-IP with a K48-linked ubiquitin-specific antibody. Results showed that PPP2R2C overexpression significantly reduced K48-linked ubiquitination of RPS27L in C666-1 and HONE1 cells, and this inhibitory effect was independent of IR treatment (Figs. 6N and S5J). These findings directly clarify that PPP2R2C suppresses the proteasomal degradation of RPS27L by inhibiting its K48-linked ubiquitination.

Collectively, these data establish that PPP2R2C promotes radioresistance in NPC cells through a precise post-translational regulatory cascade: PPP2R2C inhibits K48-linked ubiquitination of RPS27L (independent of global proteasome activity), thereby suppressing its proteasomal degradation, stabilizing RPS27L protein, and ultimately inhibiting ferroptosis.

### PPP2R2C inhibits ferroptosis and promotes radioresistance in NPC cells in vivo

**Fig. 7:**
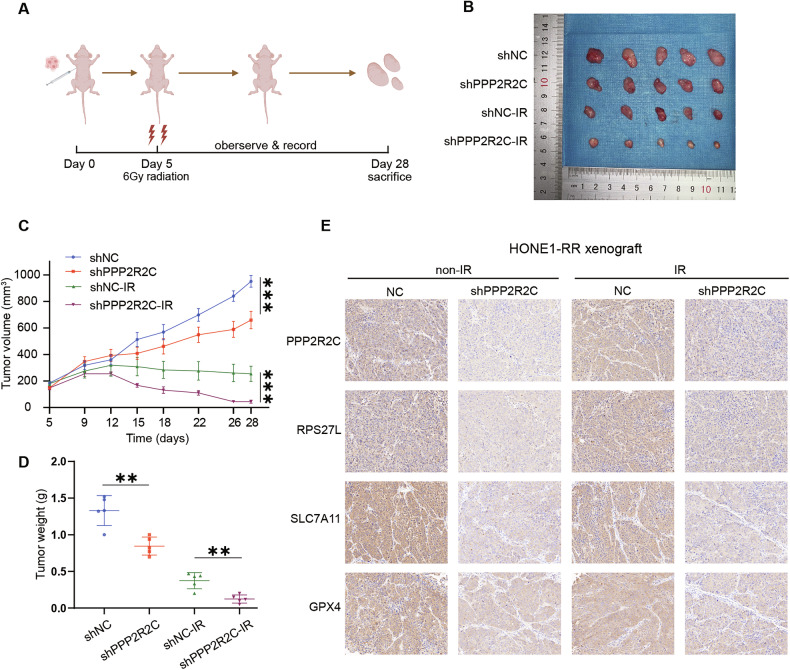
PPP2R2C promotes radioresistance by inhibiting ferroptosis in vivo. **A** Schematic of HONE1-RR xenograft establishment and irradiation timeline. **B** Tumor size, **C** volume, and **D** weight in control, IR-only, shPPP2R2C-only, and shPPP2R2C+IR group. *n* = 5 per group. Data are shown as mean ± SD. **E** Representative IHC staining of the PPP2R2C, RPS27L, SLC7A11, and GPX4 in xenografts. Scale bars: 40 μm. IR, irradiation. ^*^P < 0.05, ^**^P < 0.01, ^***^P < 0.001.

To investigate the impact of PPP2R2C on radioresistance in vivo, we subcutaneously injected HONE1 cells and stable PPP2R2C-knockdown HONE1 cells into nude mice to establish xenograft tumors. On day 5 after implantation, tumor-bearing mice received 6 Gy IR (Fig. 7A). On day 28, mice were euthanized (Fig. 7B), and tumors were dissected for tumor volume and weight measurement and immunohistochemical (IHC) staining. PPP2R2C knockdown significantly reduced subcutaneous tumor volume and weight with or without IR (Fig. 7C, D), and irradiated tumors were smaller in both volume and weight than non-irradiated counterparts. IHC staining revealed decreased expression of RPS27L, SLC7A11, and GPX4 following PPP2R2C knockdown, regardless of IR, consistent with our in vitro results (Fig. 7E). Collectively, these data demonstrate that PPP2R2C promotes radioresistance by stabilizing the RPS27L protein to inhibit ferroptosis in vivo.

## Discussion

Radiotherapy remains a cornerstone treatment for patients with NPC. However, the development of radioresistance in tumor cells contributes to local recurrence or distant metastasis in a subset of patients [4, 36]. Although current studies on radioresistance mechanisms have implicated diverse regulatory processes [10–12], these findings remain insufficient to fully elucidate or overcome this critical clinical challenge [37]. Therefore, dissecting the molecular mechanisms underlying radioresistance is pivotal for developing novel and effective radiosensitizers. Using mRNA sequencing data from paired parental and radioresistant NPC cells, together with three public datasets, we identified PPP2R2C as a key mediator of radioresistance in NPC. PPP2R2C encodes the B55γ regulatory subunit of PP2A [25–29, 38], a heterotrimeric complex comprising scaffold (A), regulatory (B), and catalytic (C) subunits [22]. Although long regarded as a tumor suppressor [39], emerging evidence indicates PP2A can exert tumor‑promoting activities in a context‑dependent manner [39–42]. This functional duality stems from its combinatorial subunit composition: whereas the A and C subunits are highly conserved, the diverse B subunit repertoire dictates substrate specificity and subcellular localization [24, 43–46].

Therapeutic targeting of PP2A remains challenging due to incomplete characterization of B-subunit functions. Although the PP2A-C inhibitor LB100 exhibits promising activity both in clinical trials (in combination with taxanes; NCT03027388, NCT04560972) [47] and preclinical studies, including synergistic effects with PD-1 inhibitors [48–50] and radiosensitization through homologous recombination inhibition [51], its limited selectivity raises significant toxicity concerns. This limitation underscores the therapeutic potential of B-subunit-specific targeting strategies. Importantly, our study demonstrates that PPP2R2C promotes radioresistance in NPC cells both in vitro and in vivo. Elucidating the PPP2R2C-mediated radioresistance could enable precise reversal of therapy resistance while minimizing potential off-target effects [52].

Among diverse mechanisms underlying radioresistance, dysregulation of programmed cell death (PCD) holds considerable pathological relevance [53–55]. Distinct from apoptosis, autophagy, and pyroptosis, ferroptosis—a recently characterized iron-dependent PCD modality driven by aberrant lipid peroxidation [56]—represents a critical modulator of the radiation response. Evasion of ferroptosis by tumor cells via inactivation of this pathway constitutes a key mechanism driving radioresistance in NPC [15, 19, 20]. Mechanistically, using both cellular assays and in vivo models, we demonstrated that PPP2R2C depletion activates ferroptosis and further confirmed that loss of PPP2R2C sensitizes NPC cells to radiation-induced ferroptosis. These findings establish ferroptosis as the executioner mechanism through which PPP2R2C promotes radioresistance, providing mechanistic insight into its pro-radioresistant function.

To further identify the substrate molecules of PPP2R2C, we performed mass spectrometry and identified RPS27L as a key downstream effector of PPP2R2C. RPS27L encodes an 84-amino-acid evolutionarily conserved ribosomal protein belonging to the 40S small subunit, which has been associated with radiation sensitivity in mice [32]. Through a series of cellular assays, we demonstrated its role in NPC radioresistance [30]. RPS27L is reported as a downstream target of the p53–MDM2 axis, and p53 and MDM2 have been widely implicated in ferroptosis across multiple studies [57–59]. We therefore hypothesized that RPS27L, similar to PPP2R2C, regulates cellular ferroptosis, which was subsequently validated using a panel of functional ferroptosis assays. Further mechanistic analyses revealed that PPP2R2C modulates RPS27L protein expression without altering its mRNA level, primarily by stabilizing RPS27L protein and preventing its proteasomal degradation.

**Fig. 8:**
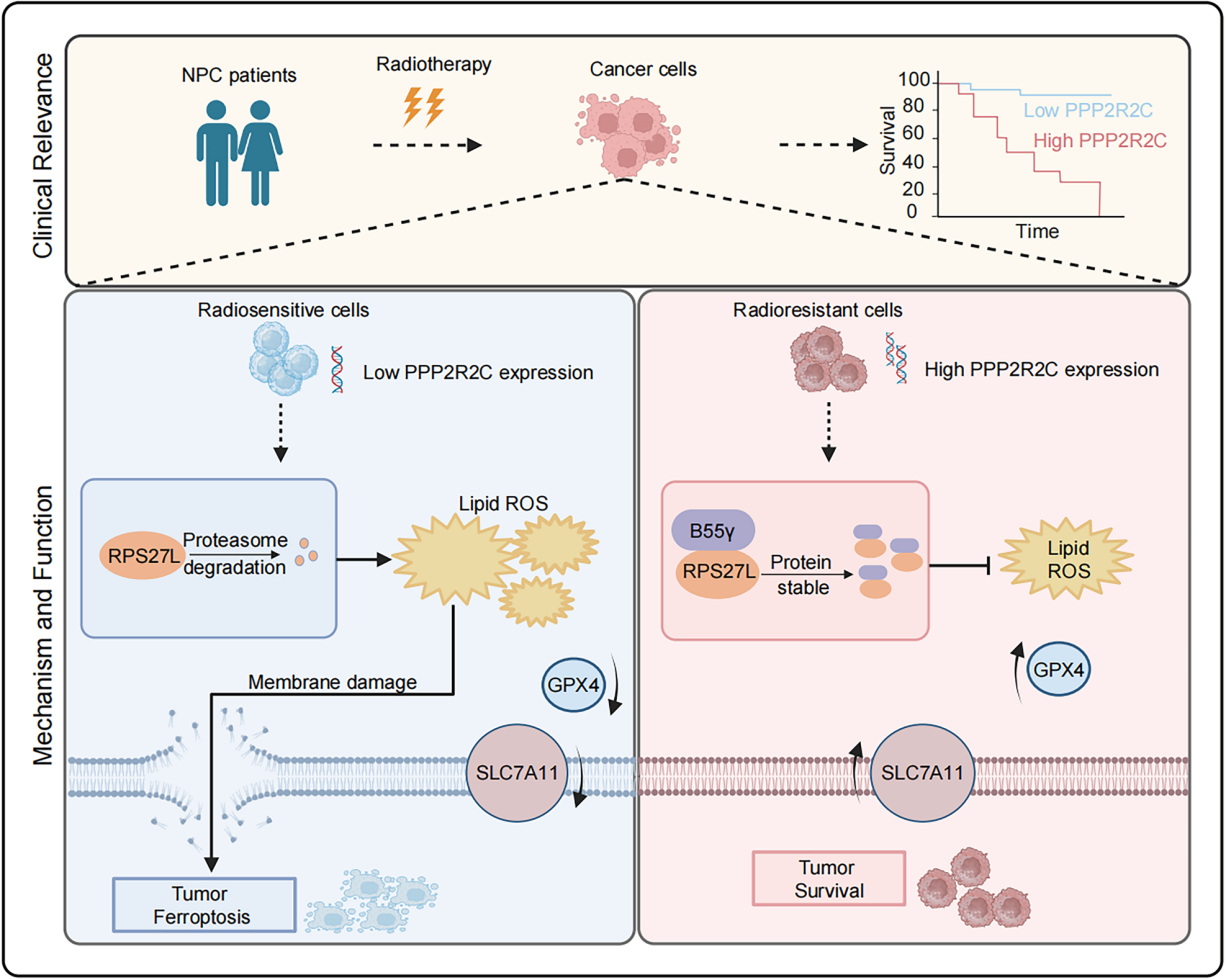
Proposed model of PPP2R2C-mediated radioresistance. PPP2R2C overexpression correlates with radioresistance and poor survival. PPP2R2C interacts with RPS27L, preventing its proteasomal degradation and collectively inhibiting ferroptosis to promote therapy resistance. This figure was created with BioRender (http://biorender.com/) under an academic license.

In summary, our study reveals the critical role of PPP2R2C in inhibiting ferroptosis and promoting radioresistance in NPC, thereby providing new insights for the development of highly selective PP2A-targeted inhibitors in the future. Mechanistically, PPP2R2C interacts with RPS27L, an oncogenic factor that promotes radioresistance, and protects it from proteasomal degradation, ultimately suppressing cellular ferroptosis (Fig. 8). Notably, high PPP2R2C expression in NPC patients is associated with poor radiotherapy prognosis. Our findings not only enable effective risk stratification for patient subgroups but also identify promising therapeutic targets for the development of novel, effective radiosensitization strategies in the future.

## Methodology

### Public datasets

Publicly available datasets were used in accordance with their respective open-access or public database terms. The public dataset GSE53819 was obtained from the Gene Expression Omnibus (GEO) database. This dataset comprises mRNA data from 18 nasopharyngeal carcinoma (NPC) tissues and 18 normal nasopharyngeal tissues. Head and neck squamous cell carcinoma (HNSC) from The Cancer Genome Atlas (TCGA) cohort was accessed via the R/Bioconductor package ‘TCGAbiolinks’ (R version 4.2.2). Following treatment-based filtering, we retained mRNA data and matched survival data for 312 HNSC patients who received radiotherapy. The Tay cohort [35], which was obtained from a previously published open-access article, provided mRNA data and corresponding survival data for 47 NPC patients treated with radiotherapy.

### Cell culture, cell lines establishment and cell transfection

Human NPC cell lines C666-1 and HONE1 were purchased from Qunxian Biotechnology (Guangzhou, China) and were additionally identified using short tandem repeats (STR) profiling. Radioresistant NPC cell lines C666-1-RR and HONE1-RR were constructed in our previous study by exposing parental C666-1 and HONE1 cells to fractionated radiation and were additionally identified using STR profiling [34]. Cells were cultured in RPMI 1640 medium supplemented with 10% fetal bovine serum at 37 °C in a humidified incubator with 5% CO₂. Cell cultures were regularly controlled for Mycoplasma negativity using the MycoAlert Detection Kit (Lonza).

For transient transfection experiments, small interfering RNA (siRNA) oligonucleotides (Hanyi, Shenzhen, China) or plasmid DNAs (Hanyi, Shenzhen, China) were transfected into the cells using Lipofectamine 3000 and OPTI-MEM (Invitrogen, Inchinnan, UK) according to the manufacturer’s instructions. For a stable transfection experiment, lentivirus encoding shPPP2R2C, noncoding shRNA (shNC), overexpression PPP2R2C, or overexpression control (Vector) was transduced into NPC cells according to instructions. The infection efficiency was verified by RT-qPCR and Western blotting. The sequences of the shRNAs and siRNAs are shown in Table S1.

### Transwell migration assay

Cell migration was assessed using Transwell chambers. An appropriate number of cells in 200 μL serum-free medium were seeded in the upper chambers, while lower chambers contained 10% FBS-supplemented medium. After 16 h incubation, non-migrated cells were removed from the upper surface. Migrated cells on the lower membrane surface were fixed in 100% methanol and stained with 0.1% crystal violet. Migration was quantified by microscopic visualization.

### Cell clonogenic capacity assay and clonogenic survival assay

The clonogenic capacity assay was performed by plating NPC cells into 6-well plates at appropriate densities. Following incubation for 7–10 days to allow colony formation, the cells were rinsed with PBS and fixed with 4% paraformaldehyde for 15 min. Fixed colonies were stained with 0.5% crystal violet (Macklin, Shanghai, China) for 30 min. Only colonies containing ≥50 cells were counted to determine the surviving fraction. For the clonogenic survival assay, cells seeded in 6-well plates were then exposed to different doses of ionizing radiation or drug treatment. After colony staining and counting, the dose-survival relationship was modeled using the multi-target single-hit equation: SF = 1–(1–*e*^[−*k*D]^) *N*. The radiobiological parameters were derived: *D*_0_ = 1/*k*, Dq = *D*_0_ × $\mathrm{ln}$ (*N*).

### Cell proliferation assay and cell viability assay

NPC cells were seeded into 96-well plates at an appropriate density and cultured for 24 h to allow attachment. For the cell proliferation assay, the medium was replaced with 100 μL of fresh medium containing 10% Cell Counting Kit-8 (CCK-8) reagent on days 0, 1, 2, 3, and 4. After 1–4 h of incubation (optimized based on cell type), absorbance at 450 nm was measured using a microplate reader (Bio-Rad, USA). A cell proliferation curve was then plotted using the mean values of triplicate wells. For the cell viability assay, cells were irradiated with 4 Gy ionizing radiation. Following 24–48 h of post-IR incubation, the medium was replaced with 100 μL of CCK-8-containing medium. Absorbance was measured similarly, and cell viability was calculated according to the manufacturer’s protocol.

### RNA extraction and quantitative reverse transcription PCR (RT-qPCR)

Total RNA was isolated from NPC cells using TRIzol reagent (Invitrogen, Inchinnan, UK) following the manufacturer’s instructions. RNA concentrations were measured with a Nanodrop 2000 (Thermo Fisher, Dartford, UK). RT-qPCR was performed on an ABI Illumina instrument (Foster, Connecticut, USA) using SYBR Green (Tiangen, Beijing, China), and relative mRNA expression was analyzed via the 2^-ΔΔCT^ method. Primer sequences are listed in Table S2.

### High-throughput RNA sequencing and data processing

Total RNA was extracted from cell samples using TRIzol reagent. RNA purity was assessed using the NanoDrop 2000/8000 microspectrophotometer, while concentration and integrity were measured using the Agilent 4200 TapeStation system with Agilent RNA ScreenTape Assay. Samples passing quality control proceeded to library preparation and sequencing. Raw sequencing data underwent quality control and filtering using fastp (version 0.12.4). Filtered reads were then aligned to the human reference genome (GRCh38) using HISAT2 (version 2.2.1). Gene expression quantification and generation of the expression matrix were performed using featureCounts from the Rsubread package (version 2.0.0). Gene expression difference analysis was carried out using R package “edgeR”. Kaplan–Meier (KM) survival analysis was performed with R package “survival”. Gene enrichment analysis was conducted using R package ‘clusterProfiler’. All statistical analyses were performed in R (version 4.2.2).

### Western blotting

NPC cells were harvested, washed with cold PBS, and lysed in RIPA buffer (Biyuntian, Shanghai, China) at 4 °C for 30 min. Protein concentrations were determined via BCA assay. Equal protein amounts were separated by SDS-PAGE and transferred to PVDF membranes. Membranes were blocked with 5% bovine serum albumin, incubated with primary antibodies overnight at 4 °C, followed by horseradish peroxidase-conjugated secondary antibodies for 1–2 h. Protein bands were visualized using enhanced chemiluminescence. Antibody details are provided in Table S3.

### Co-immunoprecipitation (Co-IP) and chromatography-mass spectrometry

Cells were lysed in IP buffer (Thermo Fisher, Dartford, UK) at 4 °C for 30 min. Lysates underwent overnight immunoprecipitation at 4 °C using anti-PPP2R2C antibody (Santa Cruz, London, UK), followed by 4 h incubation with Protein A/G agarose beads (Thermo Fisher, Dartford, UK). Immune complexes were washed three times with PBS and eluted in 2× SDS loading buffer (Invitrogen, Inchinnan, UK) for western blotting analysis with the indicated antibodies. Co-immunoprecipitates were subsequently analyzed by liquid chromatography-mass spectrometry (LC-MS; Genechem, Shanghai, China).

### Cycloheximide (CHX) assay

Cells cultured in six-well plates were pre-transfected with siRNA. After 48 h, cultures were treated with 10 μg/mL cycloheximide (CHX) and harvested at 0, 4, 8, and 12 h post treatment. Following cell lysis, protein levels were analyzed by western blotting using specified antibodies.

### Immunofluorescence (IF)

NPC cells seeded on slides overnight were fixed with 4% paraformaldehyde and permeabilized with 0.5% Triton X-100. After blocking with 5% BSA, samples were incubated with specified primary antibodies at 4 °C overnight, followed by dark incubation with fluorophore-conjugated secondary antibodies (Abcam, Cambridge, UK). Nuclei were counterstained with DAPI (Solarbio, Beijing, China), and images acquired using a confocal microscope (Nikon A1R).

### Transmission electron microscopy (TEM)

Cell samples were gently scraped in 1 mL PBS using cell scrapers and transferred to 1.5 mL tubes. Following centrifugation, pellets were fixed with 500 μL of 2.5% glutaraldehyde at room temperature. After dehydration, embedding, and ultrathin sectioning, samples were examined using a Hitachi H-7500 transmission electron microscope.

### Lipid peroxidation assay

Following treatment with irradiation (IR), cells on 6-well plates were washed twice with PBS after 24 h. Subsequently, 500 μL of PBS containing 1 μl C11-BODIPY 581/591 (Thermo Fisher, Dartford, UK) was added for 30-min dark incubation. After removing unbound dye by PBS washing, lipid peroxidation was assessed via flow cytometry (Beckman cytoflex) through simultaneous measurement of green (484/510 nm) and red (581/610 nm) fluorescence emission.

### Thiobarbituric acid (TBA) assay

Malondialdehyde (MDA) levels were quantified in NPC cells using a thiobarbituric acid (TBA) assay kit (Solarbio, Beijing, China) according to manufacturer’s specifications. Following 24-h post irradiation incubation, cells were lysed in RIPA buffer and centrifuged at 12,000 × *g* for 10 min. Subsequently, 100 μL supernatant was reacted with 200 μL TBA reagent at 95 °C for 60 min. After cooling to room temperature, absorbance was measured at 532 nm using a SpectraMax i3x microplate reader. MDA concentrations were normalized to total protein content determined by BCA assay and expressed as nmol/mg protein.

### Ferrous iron (Fe^2+^) detection

Ferrous iron (Fe^2+^) detection was performed using a colorimetric assay kit (Elabscience, Wuhan, China). Briefly, cells were collected and lysed with Reagent 1. After lysis, samples were centrifuged at 12,000 × *g* for 10 min, and the supernatant was collected and kept on ice to prevent Fe^2+^ oxidation. For microplate-based detection, 80 µL of Fe^2+^ standard solution was added to standard wells, while 80 µL of sample supernatant was added to test and control wells. Subsequently, 80 µL of Reagent 2 was added to control wells, and 80 µL of Reagent 3 was added to test and standard wells. The mixture was gently mixed and incubated at 37 °C for 10 min to form a stable Fe^2+^-chromogen complex. Absorbance values at 593 nm were measured using a microplate reader, and the Fe^2+^ concentration in samples was calculated based on the standard curve.

### Proteasome activity detection

Proteasome activity was measured using the Proteasome Activity Assay Kit (Abcam, Cambridge, UK) following the manufacturer’s instructions. Briefly, cells were lysed in NP40 lysis buffer, centrifuged at 12,000 × *g* for 10 min at 4 °C, and the supernatant was collected for protein concentration normalization via BCA assay. 50 μL of normalized cell lysate was added to a black 96-well plate, with MG132-treated lysate as inhibitor control. After 15 min pre-incubation at 37 °C, 50 μL of Succ-LLVY-AMC substrate was added, and the mixture was incubated in the dark for 30 min. Fluorescence intensity was measured at ex/em = 350/440 nm using a fluorometric microplate reader, and net proteasome activity (excluding non-specific protease activity) was calculated by subtracting inhibitor control values, expressed as relative fluorescence units (RFU) per minute per μg protein.

### Xenograft tumour models

Female BALB/c nude mice (3 weeks old; BesTest, Zhuhai, China) were randomized into treatment groups. Xenograft tumors were established by subcutaneous injection into the right posterior flank. When tumors reached 150–200 mm³, tumor-bearing mice received localized irradiation (6 Gy). Tumor volumes were monitored using the formula (width² × length)/2. Following treatment completion, mice were euthanized for tumor collection and immunostaining analysis.

### Immunohistochemical (IHC) staining

Tissue slides underwent deparaffinization in xylene followed by graded alcohol rehydration. After quenching endogenous peroxidase with 3% H₂O₂, antigen retrieval was performed by steaming in 0.1 M sodium citrate buffer (pH 6.0). Sections were then blocked with 5% BSA and incubated overnight at 4 °C with primary antibodies. Secondary staining was conducted using the GTVision™ III Detection System (GeneTech, Shanghai, China). Images were acquired on an Olympus BX63 automated microscope, with staining scores evaluated based on intensity and distribution.

### Statistics

All statistical analyses were conducted using R software (version 4.3.2) and GraphPad Prism (version 8.0.2). Results are expressed as mean ± SD. Group differences were assessed by two-tailed independent Student’s t-test or one-way ANOVA with Dunnett’s multiple comparisons test, with significance defined as *P* < 0.05. Survival curves were generated via the Kaplan–Meier method, and inter-group differences evaluated using log-rank tests. Significance levels are denoted as follows: ^*^(*P* < 0.05), ^**^(*P* < 0.01), ^***^(*P* < 0.001). Unless specified otherwise, experiments were performed at least in triplicate.

## Supplementary information

Figure S1

Figure S2

Figure S3

Figure S4

Figure S5

Supplementary legends

Tables

Original blots

## Data Availability

Source data and reagents are available from the corresponding author upon reasonable request.
