# Supplementary figures and images for: PPP2R2C confers radioresistance in nasopharyngeal carcinoma by suppressing ferroptosis via RPS27L stabilization
Source: Cell Death Dis. 2026 May 11;17(1):587. doi: 10.1038/s41419-026-08732-y (PMC13282407; doi:10.1038/s41419-026-08732-y)

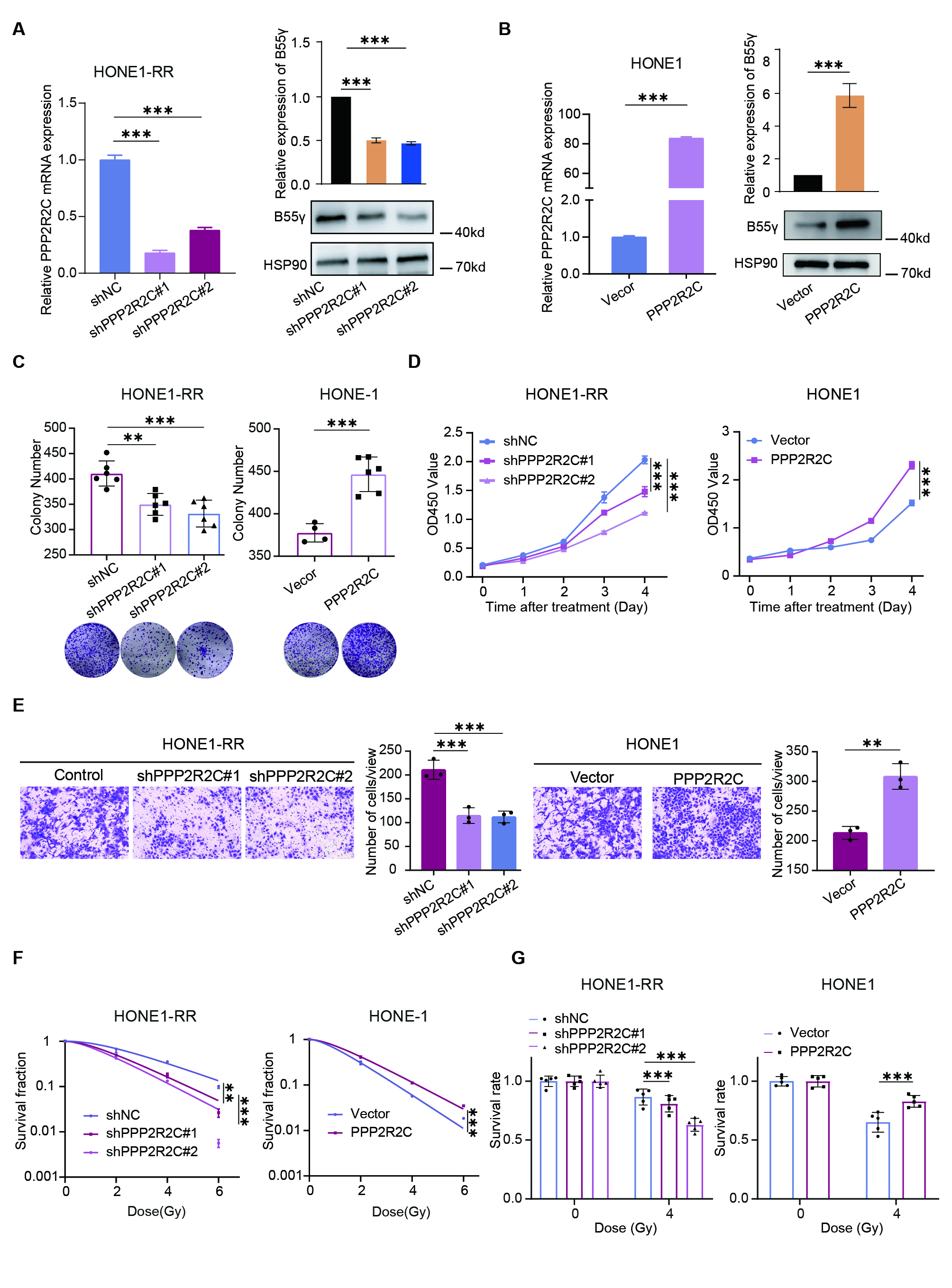

Supplement: Supplementary file 1 — Figure S1 [file 41419_2026_8732_MOESM1_ESM.tif]

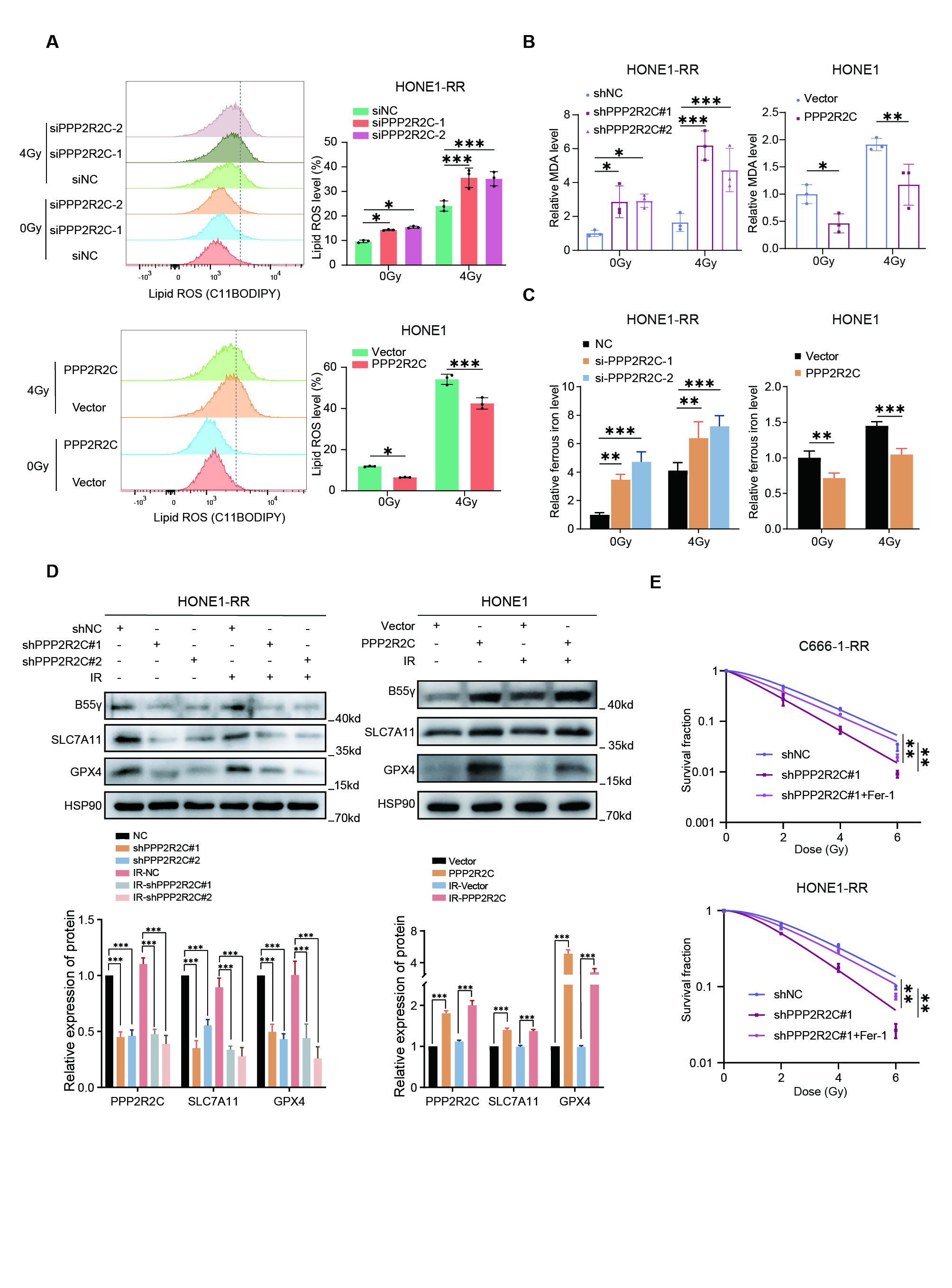

Supplement: Supplementary file 2 — Figure S2 [file 41419_2026_8732_MOESM2_ESM.tif]

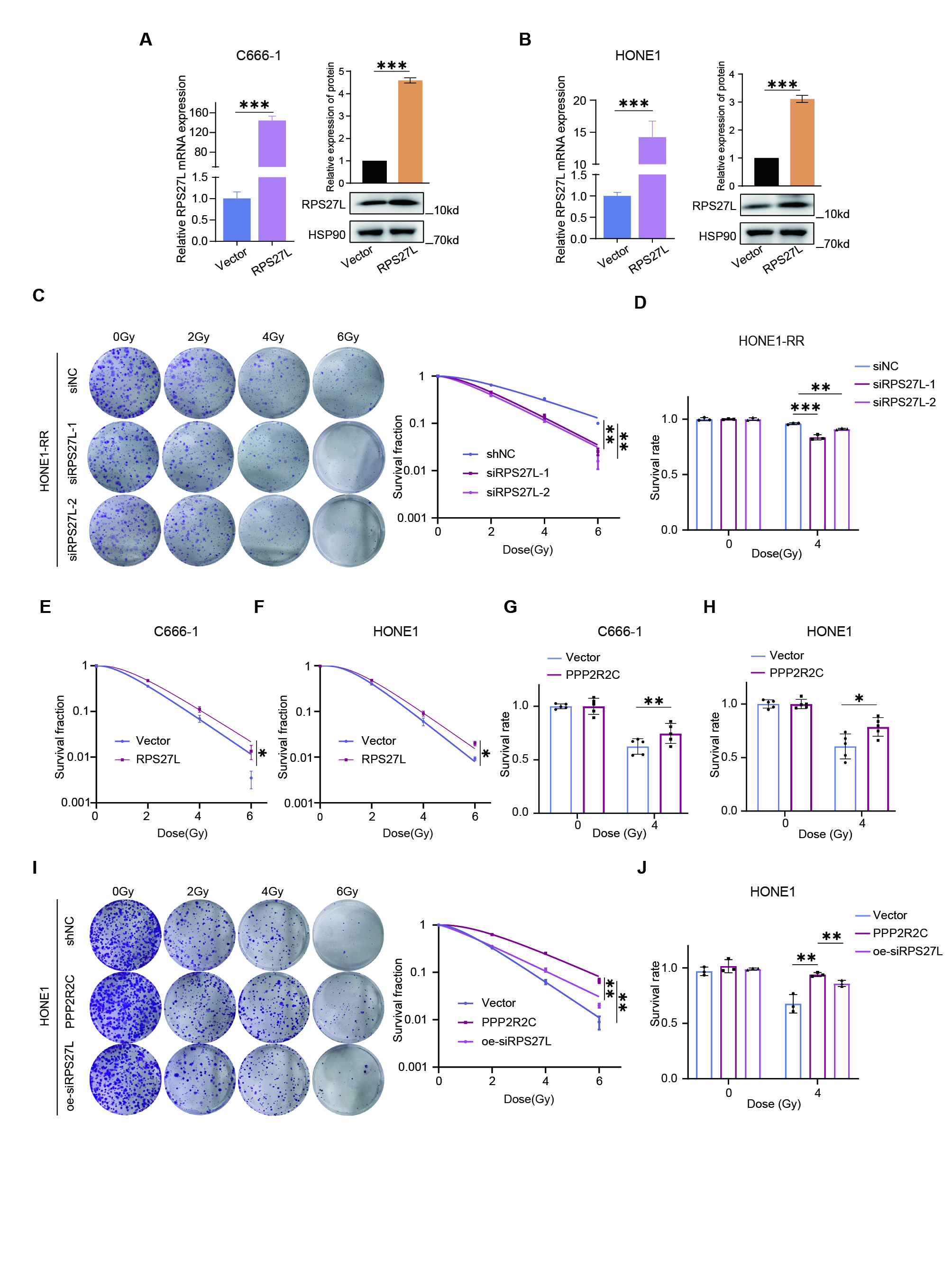

Supplement: Supplementary file 3 — Figure S3 [file 41419_2026_8732_MOESM3_ESM.tif]

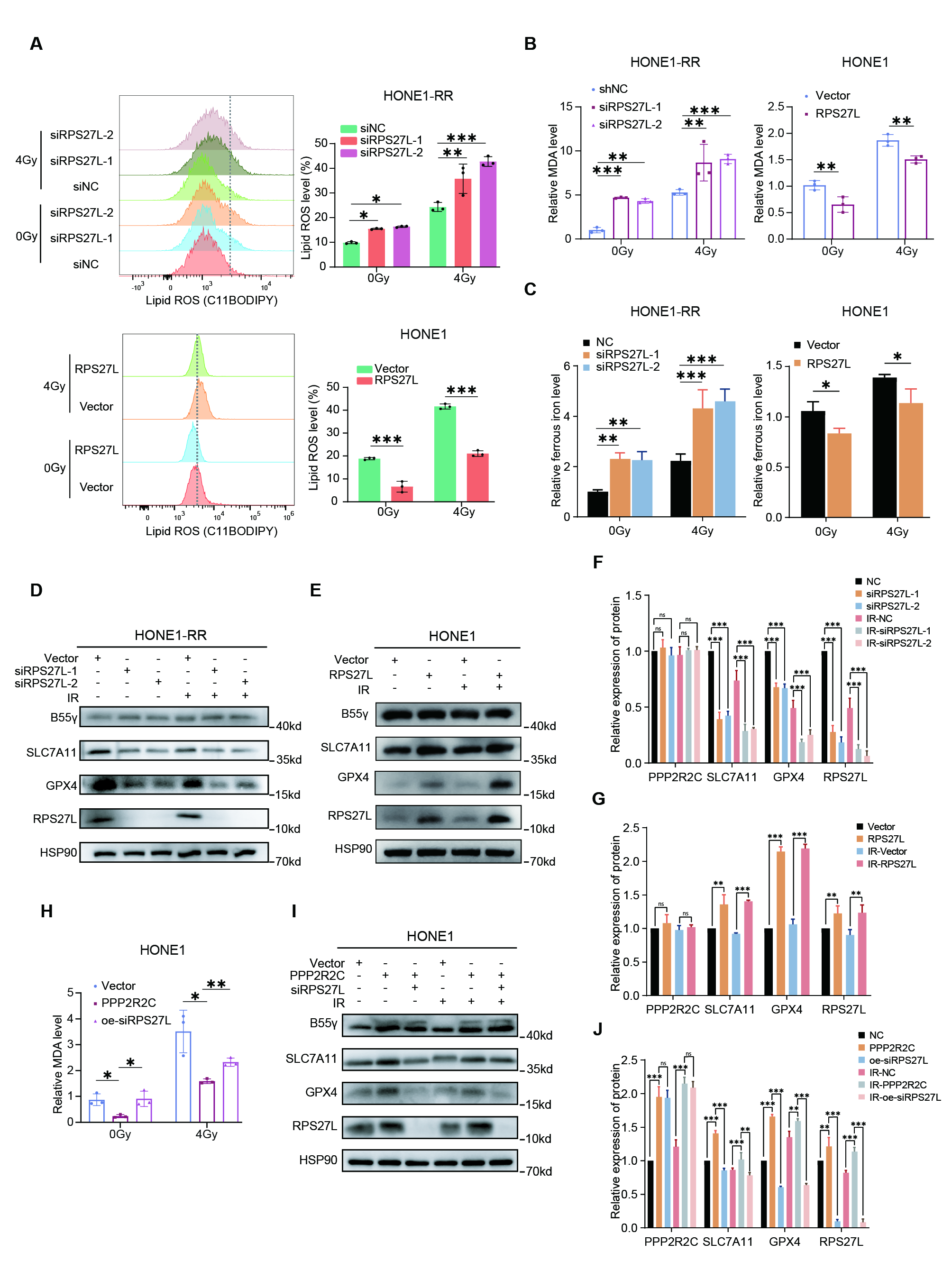

Supplement: Supplementary file 4 — Figure S4 [file 41419_2026_8732_MOESM4_ESM.tif]

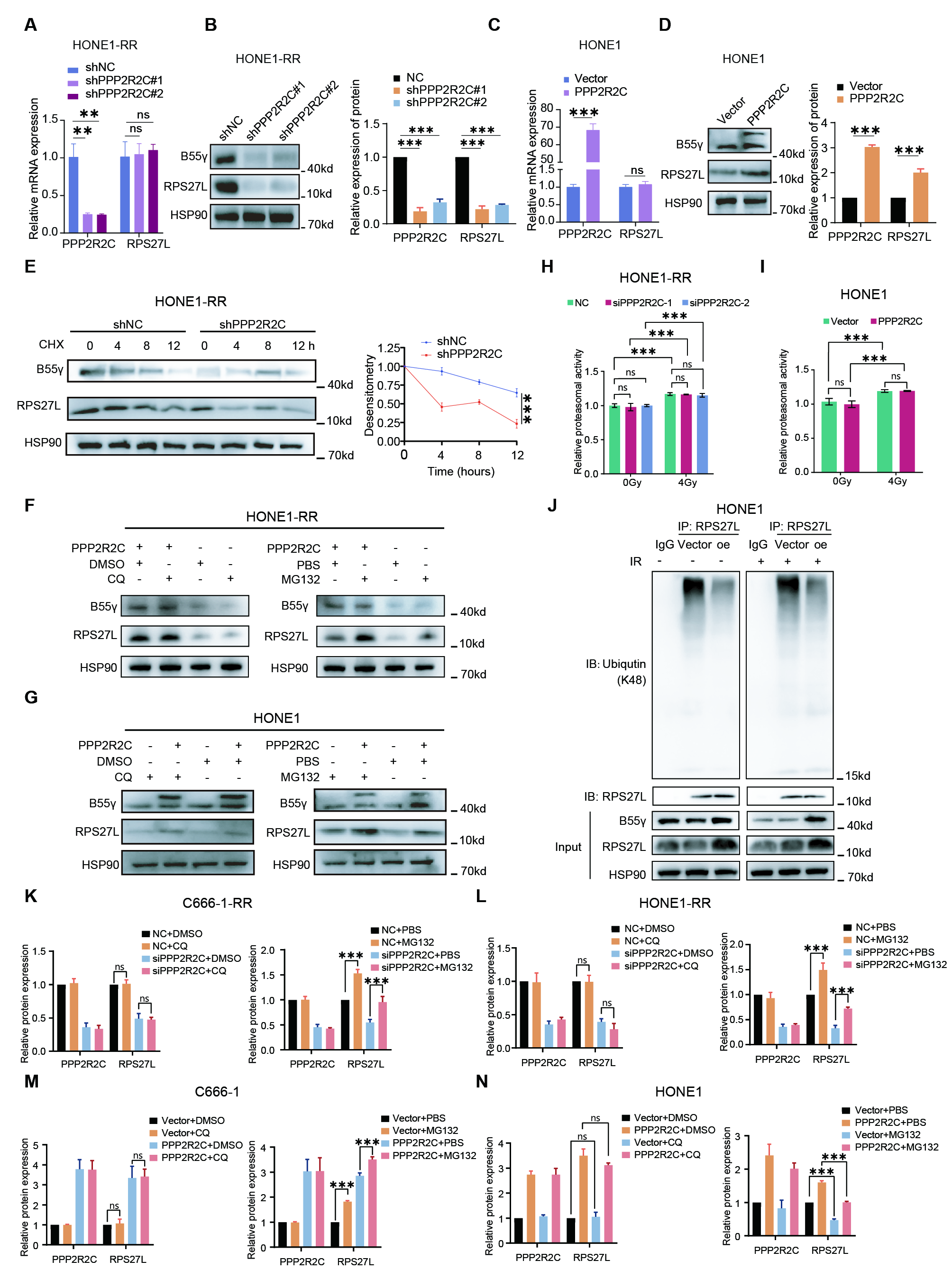

Supplement: Supplementary file 5 — Figure S5 [file 41419_2026_8732_MOESM5_ESM.tif]
