# Supplementary material for: PPP2R2C confers radioresistance in nasopharyngeal carcinoma by suppressing ferroptosis via RPS27L stabilization
Source: Cell Death Dis. 2026 May 11;17(1):587. doi: 10.1038/s41419-026-08732-y (PMC13282407; doi:10.1038/s41419-026-08732-y)
Supplement: Supplementary file 6 — Supplementary legends [file 41419_2026_8732_MOESM6_ESM.docx]

**Figure S1. PPP2R2C drives malignant phenotypes and radioresistance.** **(A)** Reduced PPP2R2C mRNA/protein post-knockdown in HONE1-RR cells. n = 3 per group. Data are shown as mean ± SD. **(B)** Increased PPP2R2C mRNA/protein after overexpression in HONE1 cells. n = 3 per group. Data are shown as mean ± SD. **(C)** Reduced colony formation with PPP2R2C knockdown in HONE1-RR cells; increased formation with overexpression in HONE1 cells. n = 6 per group. Data are shown as mean ± SD. **(D)** Impaired proliferation with PPP2R2C knockdown in HONE1-RR cells; enhanced proliferation with overexpression in HONE1 cells. n = 3 per group. Data are shown as mean ± SD. **(E)** Decreased migration with PPP2R2C knockdown in HONE1-RR cells; increased migration with overexpression in HONE1 cells. n = 3 per group. Data are shown as mean ± SD. **(F)** Reduced radioresistance with PPP2R2C knockdown in HONE1-RR cells; increased radioresistance with overexpression in HONE1 cells. n = 3 per group. Data are shown as mean ± SD. **(G)** Decreased post-4 Gy IR viability with PPP2R2C knockdown in HONE1-RR cells; increased viability with overexpression in HONE1 cells. n = 3 per group. Data are shown as mean ± SD. IR, irradiation. Statistical significance was evaluated using two-tailed independent Student’s t-test or one-way ANOVA with Dunnett’s multiple comparisons test. **P* < 0.05, ***P* < 0.01, ****P* < 0.001.

**Figure S2. PPP2R2C inhibits ferroptosis to promote radioresistance.** **(A–C)** Lipid ROS **(A)**, MDA **(B)** and intracellular ferrous iron **(C)** measurements in PPP2R2C-knockdown HONE1-RR cells and PPP2R2C-overexpressing HONE1 cells. n = 3 per group. Data are shown as mean ± SD. **(D)** Changes in protein expression of SLC7A11 and GPX4 in PPP2R2C-knockdown HONE1-RR cells and PPP2R2C-overexpressing HONE1 cells with or without IR. n = 3 per group. Data are shown as mean ± SD. **(E)** Fer-1 rescued PPP2R2C knockdown-induced radiosensitivity in C666-1-RR and HONE1-RR cells. n = 3 per group. Data are shown as mean ± SD. IR, irradiation. ROS, reactive oxygen species. MDA, malondialdehyde. Fer-1, ferroptosis inhibitor ferrostatin-1. Statistical significance was evaluated using two-tailed independent Student’s t-test or one-way ANOVA with Dunnett’s multiple comparisons test. **P* < 0.05, ***P* < 0.01, ****P* < 0.001.

**Figure S3. PPP2R2C-RPS27L interaction mediates radioresistance.** **(A–B)** Efficient RPS27L overexpression at mRNA/protein levels in **(A)** C666-1 cells and **(B)** HONE1 cells. n = 3 per group. Data are shown as mean ± SD. **(C–D)** RPS27L knockdown impaired **(C)** clonogenic survival and **(D)** viability following 4 Gy IR in HONE1-RR cells. n = 3 per group. Data are shown as mean ± SD. **(E–H)** RPS27L overexpression increased **(E–F)** clonogenic survival and **(G–H)** viability following 4 Gy IR in C666-1 cells and HONE1 cells. n = 3 per group. Data are shown as mean ± SD. **(I–J)** RPS27L knockdown reversed PPP2R2C-overexpression-induced radioresistance in HONE1 cells. n = 3 per group. Data are shown as mean ± SD. IR, irradiation. Statistical significance was evaluated using two-tailed independent Student’s t-test or one-way ANOVA with Dunnett’s multiple comparisons test. **P* < 0.05, ***P* < 0.01, ****P* < 0.001.

**Figure S4. PPP2R2C inhibits ferroptosis via RPS27L.** **(A)** Increased lipid ROS levels in RPS27L-knockdown HONE1-RR cells ± 4 Gy IR and decreased lipid ROS levels in RPS27L-overexpressing HONE1 cells ± 4 Gy IR. n = 3 per group. Data are shown as mean ± SD. **(B)** Increased MDA levels in RPS27L-knockdown HONE1-RR cells ± 4 Gy and decreased MDA levels in RPS27L-overexpressing HONE1 cells ± 4 Gy. n = 3 per group. Data are shown as mean ± SD. **(C)** Increased ferrous iron levels in RPS27L-knockdown HONE1-RR cells ± 4 Gy IR and decreased ferrous iron levels in RPS27L-overexpressing HONE1 cells ± 4 Gy IR. n = 3 per group. Data are shown as mean ± SD. **(D)** Western blot of SLC7A11/GPX4 protein expression after RPS27L knockdown ± IR in HONE1-RR cells. **(E)** Western blot of SLC7A11/GPX4 protein expression after RPS27L overexpression ± IR in HONE1 cells. **(F)** Statistical analyses of SLC7A11/GPX4 protein expression after RPS27L knockdown ± IR in HONE1-RR cells. n = 3 per group. Data are shown as mean ± SD. **(G)** Statistical analyses of SLC7A11/GPX4 protein expression after RPS27L overexpression ± IR in HONE1 cells. n = 3 per group. Data are shown as mean ± SD. **(H–J)** Knockdown of RPS27L reversed the decreased **(H)** MDA accumulation and increased **(I–J)** SLC7A11/GPX4 expression induced by PPP2R2C overexpression. n = 3 per group. Data are shown as mean ± SD. Statistical significance was evaluated using two-tailed independent Student’s t-test or one-way ANOVA with Dunnett’s multiple comparisons test. **P* < 0.05, ***P* < 0.01, ****P* < 0.001, ns: not significant.

**Figure S5. PPP2R2C stabilizes RPS27L by reducing its proteasomal degradation.** **(A–D)** RPS27L mRNA/protein levels following **(A–B)** PPP2R2C knockdown in HONE1-RR cells and **(C–D)** PPP2R2C overexpression in HONE1 cells. n = 3 per group. Data are shown as mean ± SD. **(E)** Cycloheximide (CHX) chase assay showed that PPP2R2C stabilized RPS27L protein in HONE1-RR cells. n = 3 per group. Data are shown as mean ± SD. **(F–G)** Effects of chloroquine (CQ) and MG132 on RPS27L stability in **(F)** PPP2R2C-knockdown HONE1-RR cells and **(G)** PPP2R2C-overexpressing HONE1 cells. n = 3 per group. Data are shown as mean ± SD. **(H–I)** Changes in proteasomal activity in **(H)** HONE1-RR cells upon PPP2R2C knockdown and **(I)** HONE1 cells upon PPP2R2C overexpression. n = 3 per group. Data are shown as mean ± SD. **(J)** Ubiquitination (K48) assay of RPS27L performed in PPP2R2C-overexpressing HONE1 cells. **(K)** Statistical analyses of RPS27L protein expression in PPP2R2C-knockdown C666-1-RR cells treated with CQ or MG132. n = 3 per group. Data are shown as mean ± SD. **(L)** Statistical analyses of RPS27L protein expression in PPP2R2C-knockdown HONE1-RR cells treated with CQ or MG132. n = 3 per group. Data are shown as mean ± SD. **(M)** Statistical analyses of RPS27L protein expression in PPP2R2C-overexpression C666-1 cells treated with CQ or MG132. n = 3 per group. Data are shown as mean ± SD. **(N)** Statistical analyses of RPS27L protein expression in PPP2R2C-overexpression HONE1 cells treated with CQ or MG132. n = 3 per group. Data are shown as mean ± SD. CHX, cycloheximide. CQ, chloroquine. IR, irradiation. oe, PPP2R2C overexpression. Statistical significance was evaluated using two-tailed independent Student’s t-test or one-way ANOVA with Dunnett’s multiple comparisons test. **P* < 0.05, ***P* < 0.01, ****P* < 0.001, ns: not significant.

**Table S1. List of siRNA and shRNA sequences used in this study.**

**Table S2. List of primers used in this study.**

**Table S3. List of antibodies used in this study.**
