# Supplementary material for: PPP2R2C confers radioresistance in nasopharyngeal carcinoma by suppressing ferroptosis via RPS27L stabilization
Source: Cell Death Dis. 2026 May 11;17(1):587. doi: 10.1038/s41419-026-08732-y (PMC13282407; doi:10.1038/s41419-026-08732-y)
Supplement: Supplementary file 7 — Tables [file 41419_2026_8732_MOESM7_ESM.docx]

Table S1. List of siRNA and shRNA sequences used in this study.

| siRNA sequences | Sequence (5’ to 3’) |
| --- | --- |
| siPPP2R2C-1 | UGACAUCAUCUCUACCGUUTT |
| siPPP2R2C-2 | GCUCAUUCUUCUCGGAAAUTT |
| siRPS27L-1 | GCUACAAGAUCACCACGGUUUTT |
| siRPS27L-2 | AGGGUGUUCAUUUAGAAGAAATT |
| shRNA sequences |  |
| shPPP2R2C#1 | CTGTACGAGAACGACTGCATT |
| shPPP2R2C#2 | CCGCTCATTCTTCTCGGAAAT |

Table S2. List of primers used in this study.

| Gene | Sequence (5’ to 3’) |
| --- | --- |
| PPP2R2C-F | CACTCCTGTCCACCAACGATA |
| PPP2R2C-R | CATTGGCAAAGATCCTCCGAG |
| RPS27L-F | TTACTACATCCGTCCTTGGAAGA |
| RPS27L-R | GCATGGCTGAAAACCGTGG |

Table S3. List of antibodies used in this study.

| Antibody | Company | Catalog Number |
| --- | --- | --- |
| HSP90 | Proteintech | 60318-1-Ig |
| PPP2R2C | Proteintech | 12747-1-AP |
| PPP2R2C | Santa Cruze | 100417 |
| SLC7A11 | Abcam | ab307601 |
| GPX4 | Proteintech | 82822-2-RR |
| RPS27L | Origene | TA365919 |
| Goat Anti-Rabbit IgG antibody (HRP) | Abcam | ab205718 |
| Goat Anti-Mouse IgG antibody (HRP) | Abcam | ab97240 |
| Ubiquitin (K48) | Abcam | ab140601 |
