# Supplementary material for: PPP2R2C confers radioresistance in nasopharyngeal carcinoma by suppressing ferroptosis via RPS27L stabilization
Source: Cell Death Dis. 2026 May 11;17(1):587. doi: 10.1038/s41419-026-08732-y (PMC13282407; doi:10.1038/s41419-026-08732-y)

| 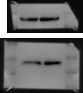Figure 2A   \| Figure 2B  HSP90  B55γ \| C666-1-RR  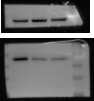 \| \| --- \| --- \|  \| Figure 2C C666-1  HSP90  B55γ \| \| --- \|  \| Figure 4D  C666-1-RR \| \| \| --- \| --- \| \| HSP90  RPS27L \| 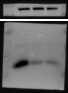 \|  \| Figure 4E \| \| \| --- \| --- \| \| HSP90  RPS27L \| HONE1-RR  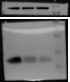 \|  \| Figure 5E C666-1 \| \| \| --- \| --- \| \| HSP90  B55γ  SLC7A11  GPX4  RPS27L \| 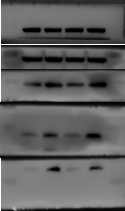 \|  \| Figure 5I  C666-1 \| \| \| --- \| --- \| \| HSP90  B55γ  SLC7A11  GPX4  RPS27L \| 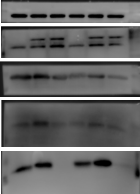 \|   HONE1-RR  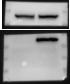  HSP90  B55γ  C666-1-RR  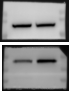 |
| --- | --- | --- | --- | --- | --- | --- | --- | --- | --- | --- | --- | --- | --- | --- | --- | --- | --- | --- | --- |

| Figure 3F  HSP90  B55γ  SLC7A11  GPX4 | C666-1-RR  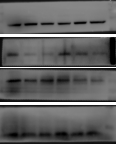 | C666-1 | |
| --- | --- | --- | --- |
|  |  | HSP90  B55γ  SLC7A11  GPX4 | 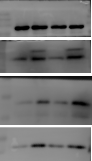 |

| Figure 4C | | | |
| --- | --- | --- | --- |
| B55γ  RPS27L  B55γ  RPS27L | C666-1-RR  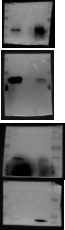 | HONE1-RR | |
|  |  | 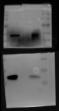  B55γ  RPS27L | |
|  |  | B55γ  RPS27L | 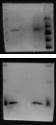 |

| Figure 5D  C666-1-RR | |
| --- | --- |
| HSP90  B55γ  SLC7A11  GPX4  RPS27L | 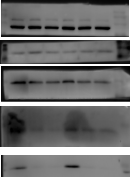 |

| 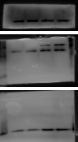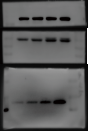Figure 6F   \| Figure 6H  HSP90  B55γ  RPS27L \| C666-1  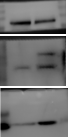 \| \| --- \| --- \|  \| Figure 6D \| \| \| --- \| --- \| \| HSP90  B55γ  RPS27L \| 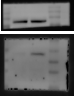  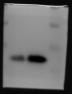 \|  \| Figure 6B \| \| \| --- \| --- \| \| HSP90  B55γ  RPS27L \| 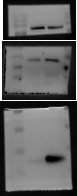 \|  \| Figure 6I  HSP90  B55γ  RPS27L \| C666-1-RR  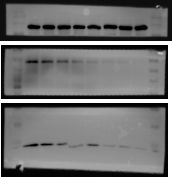 \| \| --- \| --- \|  \| Figure 6K  HSP90  B55γ  RPS27L \| C666-1  HSP90  B55γ  RPS27L \| \| --- \| --- \|   HSP90  B55γ  RPS27L | C666-1-RR  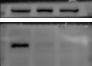  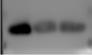 |
| --- | --- | --- | --- | --- | --- | --- | --- | --- | --- | --- | --- | --- | --- | --- | --- |

| Figure 6J  HSP90  B55γ  RPS27L | 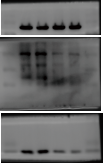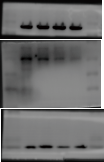C666-1-RR  HSP90  B55γ  RPS27L |
| --- | --- |

| Figure 6N | | | C666-1 | | | | |
| --- | --- | --- | --- | --- | --- | --- | --- |
| IB: Ubi quitin (K48) | | 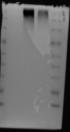 | IB: RPS27L IB: RPS27L | 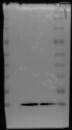 | HSP90  B55γ  RPS27L | | 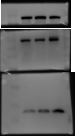 |
| IB: Ubi quitin (K48) | 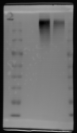 | |  | 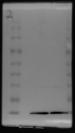 | | 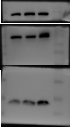HSP90  B55γ  RPS27L | |

| 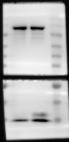Figure S2D   \| Figure S1A  HONE1-RR \| \| \| --- \| --- \| \| HSP90  B55γ \| 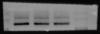  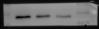 \|  \| Figure S1B  HONE1 \| \| \| --- \| --- \| \| HSP90  B55γ \| 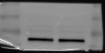  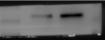 \|   HONE1   \| Figure S3B  HSP90  RPS27L \| \| --- \|  \| Figure S4I  HSP90  B55γ  SLC7A11  GPX4  RPS27L \| HONE1  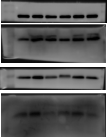  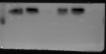 \| \| --- \| --- \|  \| Figure S4D  HSP90  B55γ  SLC7A11  GPX4  RPS27L \| HONE1-RR  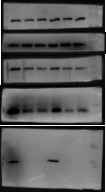 \| \| --- \| --- \|   HSP90  B55γ  SLC7A11  GPX4 | HONE1-RR  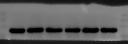  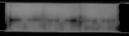  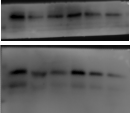 | HONE1 | |
| --- | --- | --- | --- | --- | --- | --- | --- | --- | --- | --- | --- | --- | --- | --- | --- | --- |
|  |  | HSP90  B55γ  SLC7A11  GPX4 | 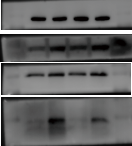 |

| Figure S3A | C666-1 |
| --- | --- |
| 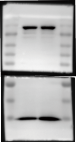  HSP90  RPS27L | |

| Figure S4E  HONE1 | |
| --- | --- |
| HSP90  B55γ SLC7A11  GPX4  RPS27L | 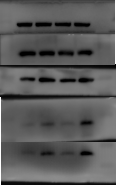 |


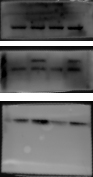

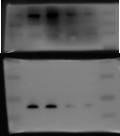

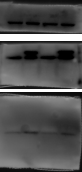


| Figure S5D  HONE1 | |
| --- | --- |
| HSP90  B55γ  RPS27L | 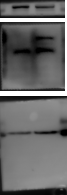 |

| Figure S5B  HONE1-RR | |
| --- | --- |
| HSP90  B55γ  RPS27L | 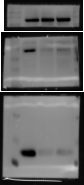 |

| Figure S5G  HSP90  B55γ  RPS27L | HSP90  B55γ  RPS27L |
| --- | --- |

| Figure S5F | | HSP90  B55γ | 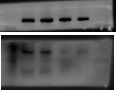 |
| --- | --- | --- | --- |
| HSP90 | 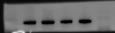 |  |  |
| B55γ  RPS27L | |  |  |
|  |  | RPS27L | |

| Figure S5E | |
| --- | --- |
| HSP90  B55γ  RPS27L | 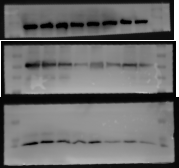 |

| Figure S5J | | | | | | | | |
| --- | --- | --- | --- | --- | --- | --- | --- | --- |
| IB: Ubi quitin (K48) | | 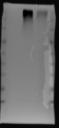 | IB: RPS27L | 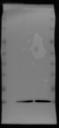 | | HSP90  B55γ | | 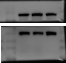 |
|  |  |  |  |  |  | 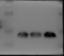  RPS27L | | |
| IB: Ubi quitin (K48) | 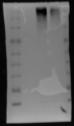 | | | IB: RPS27L | 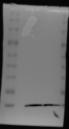 | | 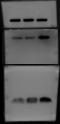  HSP90  B55γ  RPS27L | |


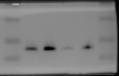

Supplement: Supplementary file 8 — Original blots [file 41419_2026_8732_MOESM8_ESM.docx]
